# Median Sternotomy Closure Using an Ultra-High-Molecular-Weight Polyethylene Suture Following Thymectomy in a Dog: A Case Report

**DOI:** 10.3390/vetsci13040311

**Published:** 2026-03-25

**Authors:** Songju Park, Jun Suk Jo, Sangyul Lee, Min-Young Kim, Hwi-Yool Kim

**Affiliations:** Department of Veterinary Surgery, College of Veterinary Medicine, Konkuk University, 120, Neungdong-ro, Gwangjin-gu, Seoul 05029, Republic of Korea; songju1015@konkuk.ac.kr (S.P.); aimingstone@naver.com (J.S.J.); sangyul7918@gmail.com (S.L.); artminky1@konkuk.ac.kr (M.-Y.K.)

**Keywords:** thymoma, median sternotomy, sternal closure, FiberWire, sternal fixation, thoracic surgery

## Abstract

A 10-year-old castrated male Chihuahua weighing 3.06 kg was presented with a chronic, progressively worsening cough of five months’ duration. Diagnostic imaging, including thoracic radiography and computed tomography, identified a well-defined cranial mediastinal mass consistent with a thymic tumor. Surgical excision was performed via median sternotomy with complete thymectomy. Following tumor removal, sternal closure was achieved using a non-absorbable ultra-high-molecular-weight polyethylene (UHMWPE) suture material (FiberWire^®^, Arthrex, Naples, FL, USA). Histopathological examination confirmed the diagnosis of an epithelial-predominant thymoma with narrow but complete surgical margins. Postoperative recovery was uneventful, and the dog was discharged three days after surgery. Clinical signs, including coughing, progressively improved during follow-up. Radiographic evaluation performed up to postoperative day 57 demonstrated stable sternal alignment without evidence of dehiscence, implant-related complications, or disease recurrence. This report describes the first clinical case of FiberWire use for median sternotomy closure following thymectomy in a dog. The favorable clinical and radiographic outcomes observed during postoperative follow-up suggest that FiberWire may represent a viable alternative to traditional stainless-steel wire for sternal fixation in canine thoracic surgery.

## Simple Summary

Thymoma is an uncommon tumor of the cranial mediastinum in dogs and is typically treated by surgical removal through median sternotomy. Closure of the sternum following this procedure is traditionally performed using stainless-steel orthopedic wire; however, wire fixation may be associated with technical challenges and postoperative complications. In this report, a 10-year-old Chihuahua was diagnosed with a cranial mediastinal thymoma and successfully treated by thymectomy via median sternotomy. The sternum was closed using a non-metallic suture material, FiberWire^®^ (Arthrex, Naples, FL, USA), instead of traditional orthopedic wire. The dog recovered uneventfully, showed progressive improvement in respiratory signs, and did not develop sternal instability or implant-related complications during radiographic follow-up. This case demonstrates that FiberWire can be a feasible alternative material for sternal closure following median sternotomy in small dogs and may help veterinarians consider additional options for improving surgical outcomes in thoracic procedures.

## 1. Introduction

Thymoma is an uncommon primary tumor of the cranial mediastinum in dogs, arising from thymic epithelial cells and typically affecting middle-aged to older patients. Although relatively rare, thymoma represents the second most common cranial mediastinal tumor in dogs and cats [1,2]. Surgical excision is considered the definitive treatment for thymomas, particularly for noninvasive tumors, and is associated with prolonged survival in dogs following complete resection [2,3]. The choice of surgical approach for cranial mediastinal masses, including thymomas, depends largely on tumor size, location, and involvement of adjacent structures [4].

Median sternotomy (MS) has been widely regarded as the approach of choice for surgical excision of cranial mediastinal masses [4,5]. However, this approach has been associated with a relatively high incidence of postoperative complications in small animals, with reported rates ranging from approximately 17% to 78% [6,7,8]. Commonly reported complications include pain, incisional swelling, seroma formation, hemorrhage, surgical site infection, sternal dehiscence, osteomyelitis, and implant failure [6,9]. Given that sternal instability is a major contributing factor to many of these complications, achieving rigid and stable fixation of the sternum is considered critical to reducing postoperative morbidity following median sternotomy [4,6,8,9].

Traditionally, stainless-steel orthopedic wire applied in a figure-of-eight configuration has been the most commonly recommended and widely used method for sternal closure in dogs. Orthopedic wire offers high tensile strength and long-term stability; however, it is also associated with several limitations, including uneven tension distribution, prolonged placement time, potential glove perforation, and risks of local tissue irritation or implant-related complications. These limitations have prompted investigation into alternative sternal closure materials and techniques aimed at improving surgical handling and postoperative outcomes [6,9,10,11].

In recent years, alternative sternal closure materials have been explored in an effort to improve handling characteristics and reduce closure-related morbidity following median sternotomy [6,9,10]. In human medicine, suture tape-based constructs have been increasingly explored for sternal closure, demonstrating favorable biomechanical properties and reduced rates of instability-related complications when compared with traditional stainless-steel wire fixation [12,13]. These constructs are typically composed of ultra-high-molecular-weight polyethylene (UHMWPE), a material shown to possess substantially higher ultimate tensile strength and superior resistance to deformation compared with conventional suture materials in biomechanical testing [10,14]. In veterinary medicine, recent biomechanical studies evaluating median sternotomy closure in dogs have demonstrated that suture tape composed of ultra-high-molecular-weight polyethylene (UHMWPE), such as FiberWire, provides mechanical stability comparable to that of orthopedic wire cerclage under distraction forces. These materials offer several practical advantages, including improved handling characteristics and avoidance of wire-related drawbacks such as wire-stick injuries and local tissue trauma [6].

Despite these encouraging experimental and biomechanical findings, clinical reports describing the application of FiberWire for median sternotomy closure in veterinary patients remain limited. To the authors’ knowledge, this report represents the first clinical case report describing the use of FiberWire for sternal closure following median sternotomy in a dog, with postoperative radiographic assessment and short-term clinical outcome evaluation. Accordingly, this case report aims to describe the surgical technique and short-term clinical outcome of median sternotomy closure using FiberWire in a dog undergoing thymectomy for a cranial mediastinal thymoma, and to provide initial clinical evidence supporting its potential use as an alternative to conventional metallic wire fixation.

## 2. Case Description

A 10-year-old castrated male long-haired Chihuahua weighing 3.06 kg was presented to the Konkuk University Veterinary Medicine Teaching Hospital with a chief complaint of a progressively worsening, non-specific cough that had been present for approximately five months. According to the owner, the cough was initially intermittent but gradually increased in both frequency and severity over time.

The patient was initially evaluated by the internal medicine service. Diagnostic evaluation included thoracic radiography and computed tomography, followed by fine-needle aspiration (FNA) of the mediastinal mass. Based on the combined diagnostic findings, a cranial mediastinal mass of suspected thymic origin was considered. The patient was subsequently referred to the surgery service, and surgical excision was elected as the treatment approach. On physical examination at the time of surgical referral, the dog was bright, alert, and responsive, and no remarkable abnormalities were identified on general examination. Cardiopulmonary auscultation revealed no significant abnormalities. However, gentle percussion applied to the mid-cervical region, slightly caudal to the larynx, consistently elicited a tracheal reflex, which was interpreted as being potentially associated with mechanical irritation of the airway secondary to the mediastinal mass. Preoperative complete blood count and serum biochemical analyses were within normal reference ranges, and no clinically significant abnormalities or concomitant diseases were identified.

**Figure 1:**
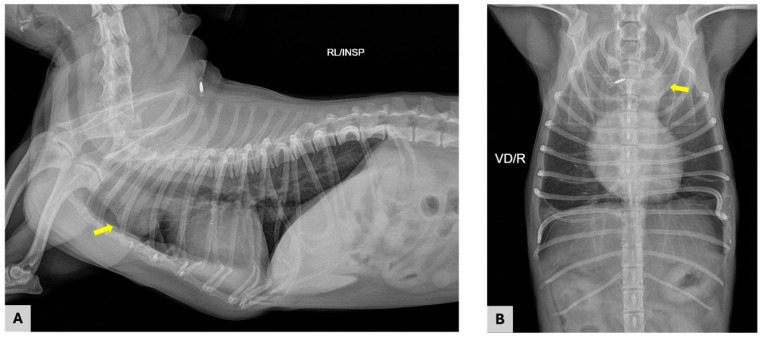
Thoracic radiographic images obtained on the day of presentation. (**A**) Right lateral view demonstrating a soft tissue opacity (arrow) in the cranial mediastinum located ventral to the trachea; (**B**) Ventrodorsal view showing a focal soft tissue opacity (arrow) within the cranial mediastinum, slightly more prominent on the left side.

Thoracic radiographs demonstrated a soft tissue opacity located in the cranial mediastinum at the level of the first to third ribs (Figure 1). On the right lateral projection, the mass was identified ventral to the trachea and resulted in mild dorsal displacement of the airway. On the ventrodorsal projection, the lesion appeared as a focal soft tissue density within the cranial mediastinum, slightly more prominent on the left side. The margins of the mass were relatively well defined, and no radiographic evidence of mineralization was observed. No abnormalities were identified within the pulmonary parenchyma, and there was no radiographic evidence of pleural effusion or pulmonary metastatic disease. Overall, these radiographic findings were consistent with the presence of a cranial mediastinal soft tissue mass, prompting further diagnostic evaluation.

Further evaluation with contrast-enhanced computed tomography (CT) was performed under general anesthesia following endotracheal intubation on the same day as thoracic radiography. CT images were acquired using a CT scanner (Aquilion Lightning; Canon Medical Systems, Otawara, Japan) following intravenous administration of iodinated contrast medium (iohexol; Omnipaque, GE Healthcare, Chicago, IL, USA).

**Figure 2:**
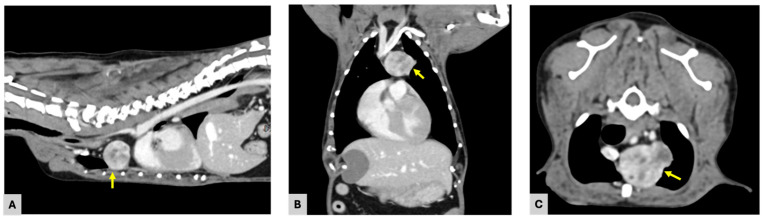
Computed tomography of the thorax demonstrating a cranial mediastinal mass: (**A**) Sagittal contrast-enhanced CT image showing a soft tissue mass in the ventral cranial mediastinum (arrow); (**B**) Dorsal contrast-enhanced CT image at the same level demonstrating the mass (arrow) in the left ventral mediastinum; (**C**) Transverse contrast-enhanced CT image showing the cranial mediastinal mass (arrow) located ventral to the trachea and slightly left of midline.

CT images revealed a well-defined, ovoid soft tissue mass located in the left ventral aspect of the cranial mediastinum, centered at the level of the first to second ribs and extending slightly caudally (Figure 2). The mass measured 25.2 × 18.6 × 20.1 mm (length × width × height) and exhibited heterogeneous contrast enhancement, with attenuation values increasing from approximately 40 Hounsfield units (HUs) on pre-contrast images to 166 HUs following contrast administration. Areas of lower attenuation consistent with focal fluid-density components were noted within the mass, contributing to the heterogeneous internal architecture of the mass.

The mass caused rightward displacement of the trachea at the level of the first to second ribs and exerted compressive effects on the cranial portion of the left cranial lung lobe. Ventrally, the lesion was closely adjacent to the left ventral thoracic wall. On its right aspect, the mass was in direct contact with the cranial vena cava. Dorsally, the mass was located in close proximity to the brachiocephalic trunk, left subclavian artery, and common carotid arteries, with an approximate distance of 1.0 mm. The distance between the mass and the ascending aorta was approximately 3.0 mm. These findings indicated close proximity to major vascular structures without definitive evidence of vascular encasement or intraluminal invasion.

Fine-needle aspiration (FNA) of the mediastinal mass was subsequently performed under ultrasound guidance prior to recovery from general anesthesia following CT. Cytologic examination revealed low cellularity with cohesive clusters of cells characterized by oval nuclei and basophilic cytoplasm, with features suggestive of mesenchymal origin. A background of erythrocytes and occasional vacuolated macrophages was noted. No cytologic features consistent with lymphoma were identified. Based on combined diagnostic findings, surgical excision via median sternotomy with thymectomy was planned. Surgical excision was performed 14 days after the CT evaluation.

On the day of surgery, general anesthesia was induced following premedication and maintained as described below. Prophylactic antimicrobial therapy was administered prior to skin incision using amoxicillin–clavulanate (13.75 mg/kg, IV; Amocla, Kuhnil Pharmaceutical, Seoul, Republic of Korea), in accordance with perioperative infection prevention guidelines. In addition, maropitant citrate (1 mg/kg, SC; Cerenia, Zoetis, Parsippany, NJ, USA) was administered preoperatively as an antiemetic. Premedication consisted of midazolam (0.2 mg/kg, IV; Bukwang Midazolam Inj., Bukwang Pharm, Seoul, Republic of Korea) and fentanyl (2 µg/kg, IV), followed by a continuous rate infusion of fentanyl (1–4 µg/kg/h, IV) for intraoperative analgesia (Fentanyl Citrate Inj., Hana Pharm, Seoul, Republic of Korea). General anesthesia was induced with propofol (6 mg/kg, IV; Anepol Inj., Hana Pharm, Seoul, Republic of Korea), followed by endotracheal intubation. Following intubation, the patient was mechanically ventilated to maintain adequate oxygenation and ventilation during the procedure. Anesthesia was maintained with isoflurane (Terrell Solution, Kyongbo Pharm, Asan, Republic of Korea) in oxygen.

Following induction of general anesthesia, the patient was positioned in dorsal recumbency and the ventral thoracic region was aseptically prepared for surgery. A ventral midline skin incision was made over the sternum, and the underlying pectoral musculature was bluntly and sharply dissected to expose the sternum.

A median sternotomy was performed by transecting the midline of the second to fourth sternebrae to expose the thoracic cavity, while the manubrium and xiphoid process were left intact. The sternotomy was conducted in a shallow and controlled manner to minimize the risk of iatrogenic injury to adjacent structures, including the cranial vena cava, brachiocephalic vein, and internal thoracic vessels. Gelpi retractors were then carefully placed to maintain gentle retraction and provide adequate exposure of the cranial mediastinum and intrathoracic structures.

**Figure 3:**
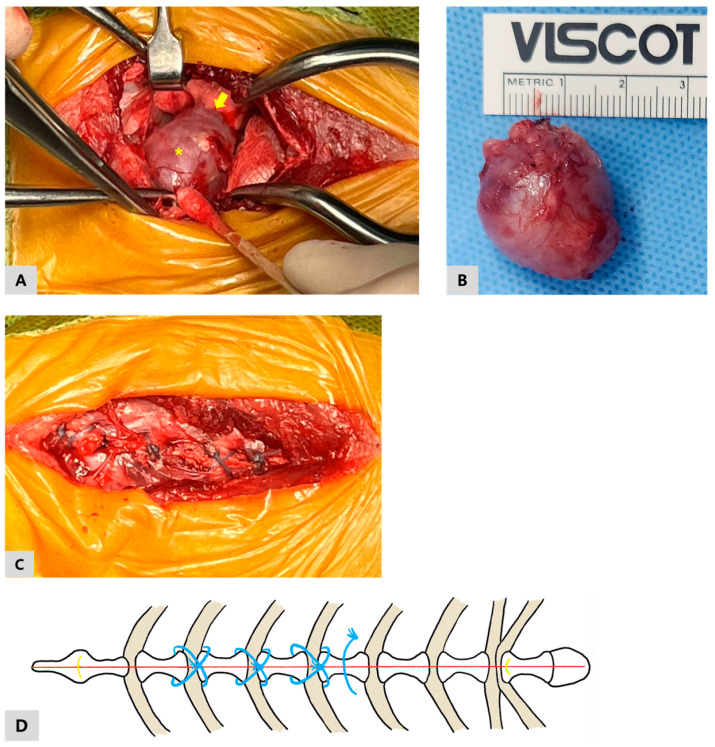
Intraoperative and gross pathologic images: (**A**) Intraoperative view following median sternotomy demonstrating a cranial mediastinal mass (asterisk) with adhesions to the left cranial lung lobe (arrow); (**B**) Gross appearance of the resected cranial mediastinal mass following thymectomy; (**C**) Intraoperative photograph showing sternal closure using an ultra-high-molecular-weight polyethylene-based suture material (FiberWire); (**D**) Schematic illustration of the sternal closure technique showing three peristernal figure-of-eight sutures placed sequentially around the sternebrae and an additional simple interrupted reinforcement suture between the fourth and fifth sternebrae.

Upon entering the thoracic cavity, a cranial mediastinal mass was identified (Figure 3A). The mass was carefully separated from the surrounding mediastinal tissues using blunt dissection, with particular attention paid to preserving nearby vascular and neural structures. During dissection, adhesions between the mediastinal mass and the cranial portion of the left cranial lung lobe were identified. These adhesions were meticulously dissected to allow complete excision of the mass while minimizing trauma to the lung parenchyma. After removal of the mass, a bubble test was performed to assess for air leakage from the left cranial lung lobe. No evidence of air leakage was observed, confirming the integrity of the lung tissue following adhesiolysis. As no pulmonary air leakage was detected, a thoracic drain was not placed at the time of closure. Hemostasis was confirmed within the surgical field prior to closure. Prior to complete closure of the thoracic cavity, the lungs were gently inflated to facilitate evacuation of residual intrathoracic air and re-establish negative intrathoracic pressure.

Sternal closure was performed using FiberWire^®^ #2 (Arthrex, Naples, FL, USA), a non-absorbable ultra-high-molecular-weight polyethylene (UHMWPE)-based suture material. After confirming appropriate reduction and alignment of the sternebrae, peristernal figure-of-eight sutures were placed sequentially around the second through fourth sternebrae to reappose the sternal halves and restore thoracic stability (Figure 3C). Each figure-of-eight suture was tightened gradually to maintain symmetric apposition, with care taken to avoid excessive focal compression of the sternal cortex. Each suture was secured using multiple square knots to ensure stable fixation. To provide additional stabilization of the caudal aspect of the sternotomy, an additional simple interrupted FiberWire suture was placed between the fourth and fifth sternebrae (Figure 3D). Throughout suture placement, particular attention was paid to maintaining a superficial trajectory adjacent to the sternebrae and avoiding deep passage dorsal to the sternum or through the costochondral junctions, given the close proximity of the internal thoracic vessels, perforating branches, and intercostal neurovascular bundles. Following completion of sternal fixation, the surgical field was re-evaluated to confirm satisfactory alignment and stability of the sternum, and no evidence of sternal instability was detected. Routine closure of the pectoral musculature and subcutaneous tissues was performed using 3-0 polydioxanone (PDS), and the skin was closed with 3-0 nylon (Dafilon) in corresponding layers.

On gross examination, the excised mass was well circumscribed and ovoid, measuring approximately 2 × 2.5 cm, with a smooth external surface (Figure 3B).

**Figure 4:**
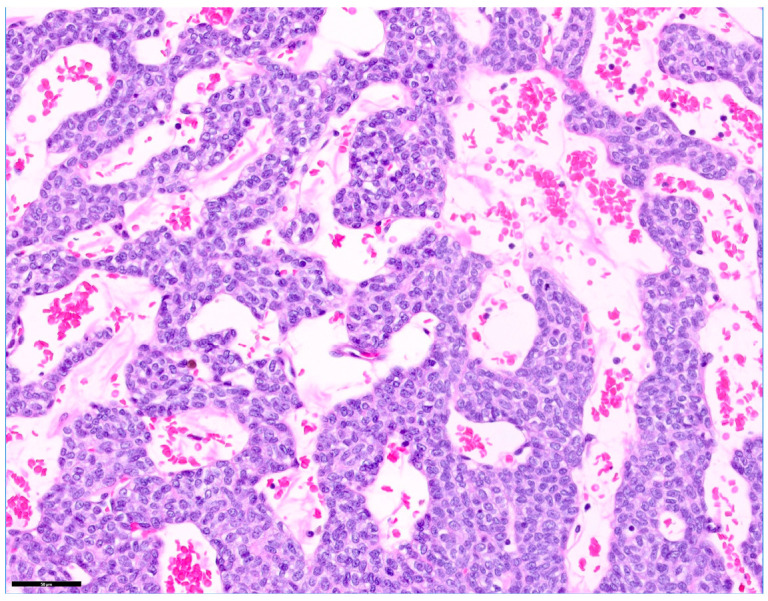
Histopathology results of the mediastinal mass: Thymoma (Hematoxylin and eosin stain, ×400).

Histopathological examination of the excised cranial mediastinal mass confirmed a diagnosis of thymoma, epithelial predominant subtype (Figure 4). The mass was composed primarily of neoplastic thymic epithelial cells with varying degrees of accompanying benign lymphocytic infiltration. The mitotic count was less than 1 per 2.37 mm^2^. No evidence of vascular invasion was identified. Surgical margins were histologically free of neoplastic cells; however, the margins were narrow, measuring less than 0.1 mm at the level of the capsule. Based on the histopathological findings, the lesion was consistent with a thymic epithelial neoplasm arising from the anterior mediastinum. The microscopic features were compatible with a well-circumscribed thymoma without definitive histologic evidence of aggressive features.

Postoperative recovery was uneventful. The patient recovered smoothly from anesthesia without evidence of respiratory compromise, wound-related complications, or other immediate postoperative concerns. Preoperative hematologic and serum biochemical analyses were within normal reference ranges. The hematocrit decreased from 41% preoperatively to 34% postoperatively but returned to within normal limits without additional intervention. Total protein decreased slightly from 6.6 g/dL preoperatively to 6.3 g/dL postoperatively and remained within normal limits throughout hospitalization. No clinically significant abnormalities were identified on postoperative serum biochemical analyses. Postoperative analgesia was provided using a continuous rate infusion of fentanyl (1–4 µg/kg/h, IV) for pain control (Fentanyl Citrate Inj., Hana Pharm, Seoul, Republic of Korea). Prophylactic antimicrobial therapy with amoxicillin–clavulanate (13.75 mg/kg, IV, q12h; Amocla, Kuhnil Pharmaceutical, Seoul, Republic of Korea) was administered postoperatively. Postoperative inflammatory response was monitored using serum C-reactive protein (CRP) concentrations. CRP levels were elevated on postoperative day (POD) 1 (8.8 mg/L) and POD 2 (9.6 mg/L), followed by a progressive decline on POD 3 (7.2 mg/L) and POD 6 (1.7 mg/L), consistent with a resolving postoperative inflammatory response. The surgical incision and sternal region were assessed daily from POD 1 through discharge on POD 3, during which mild heat, swelling, and erythema were observed without progression or signs of infection, and were considered within acceptable limits for normal postoperative healing. The dog was discharged three days after surgery in stable condition, with normal appetite and activity. During early postoperative follow-up, the frequency and severity of coughing progressively improved compared to the preoperative state. By POD 14, coughing was observed only in specific situations, such as during periods of excitement. At subsequent follow-up on POD 57, the coughing episodes were reported to be less frequent, milder, and shorter in duration than before surgery.

**Figure 5:**
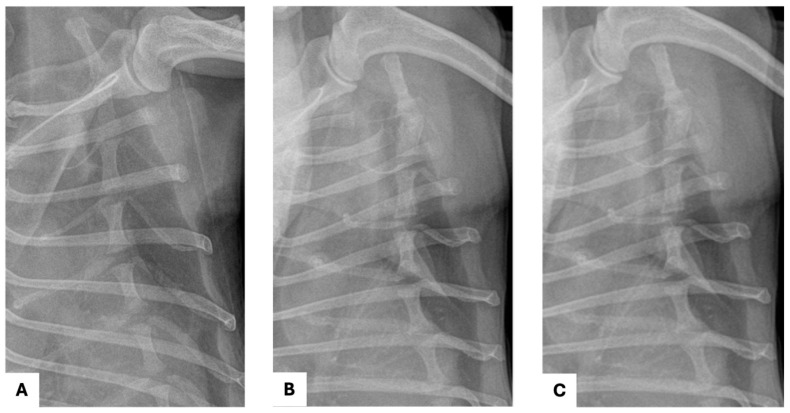
Follow-up thoracic radiographs after median sternotomy: (**A**) Magnified ventrodorsal thoracic radiograph obtained on postoperative day (POD) 10; (**B**) Magnified ventrodorsal thoracic radiograph obtained on POD 57; (**C**) Magnified ventrodorsal thoracic radiograph obtained on POD 329, demonstrating maintained sternal alignment without evidence of dehiscence or fixation loosening.

Follow-up thoracic radiographs were obtained up to POD 57 (Figure 5A,B) and demonstrated no evidence of sternal dehiscence, displacement, or other postoperative thoracic abnormalities. No radiographic signs of pleural effusion, pulmonary complications, or recurrence of the mediastinal mass were identified during the monitoring period. An additional long-term follow-up thoracic radiograph obtained on POD 329 also demonstrated maintained sternal alignment without evidence of wire loosening, displacement, or recurrence of the mediastinal mass (Figure 5C). Overall, the patient remained clinically stable throughout the follow-up period, with no major postoperative complications observed.

## 3. Discussion

Traditionally, sternal closure following median sternotomy in small animals has been performed using stainless steel orthopedic wire; however, this technique is associated with several technical and clinical limitations [6,9,10]. To the authors’ knowledge, this is the first reported canine case describing successful median sternotomy closure using FiberWire following thymectomy. In this dog, FiberWire provided stable sternal fixation without postoperative dehiscence or implant-related complications, as confirmed by serial radiographic follow-up, supporting the feasibility of FiberWire as a sternal closure material in this case.

Orthopedic wire is a metallic fixation material that concentrates compressive forces over a relatively small contact area when applied to the cortical bone of the sternebrae. This focal stress concentration may predispose to bone cut-through, particularly in small dogs or in patients with relatively thin or fragile sternal cortices, potentially resulting in postoperative sternal instability or fixation failure [3,6,9]. In contrast, FiberWire is composed of braided ultra-high-molecular-weight polyethylene (UHMWPE) fibers, which provide a broader contact interface with bone compared with traditional stainless-steel wire [6,15]. This structural characteristic allows applied loads to be distributed over a wider surface area, thereby reducing localized stress at the bone–suture interface and potentially decreasing the risk of bone cut-through [6,10,16]. Previous biomechanical studies have demonstrated that UHMWPE-based suture materials exhibit load-to-failure values comparable to those of orthopedic wire cerclage, indicating that FiberWire provides sufficient static strength to maintain sternal stability under distraction forces [6,17].

Another important biomechanical consideration is the response of fixation materials to repetitive cyclic loading generated by respiration and thoracic wall motion [3,18]. Stainless-steel wire is susceptible to metal fatigue when subjected to continuous micromotion and cyclic bending forces, which may result in wire deformation or fracture and contribute to delayed sternal instability [9,16,18]. In contrast, FiberWire is not subject to metal fatigue and therefore eliminates the risk of fatigue-related wire failure [6]. This characteristic may provide improved durability under repetitive physiological loading conditions, which is particularly relevant in sternal fixation where continuous motion is unavoidable during the postoperative period [6,9,17].

In addition to its biomechanical properties, FiberWire offers practical advantages in surgical handling [6]. Traditional stainless-steel orthopedic wire cerclage is highly technique-dependent and can be challenging to manipulate, requiring precise twisting, cutting, and bending to achieve adequate fixation [10,11,19]. Furthermore, the need for repeated wire twisting and trimming may increase operative complexity, prolong surgical time, and carry a potential risk of glove perforation or inadvertent soft tissue injury [6,10,17]. In contrast, non-metallic cerclage materials such as UHMWPE-based sutures are applied using standard knot-tying techniques and do not require specialized instruments or power tools [17]. Previous comparative and review studies have reported that non-metallic cerclage systems are generally easier to apply, less technically demanding, and more consistent in achieving secure fixation than metallic wire cerclage [9,17]. These handling characteristics may contribute to improved surgical efficiency and reduced intraoperative complexity.

From a patient comfort perspective, metallic wire cerclage has been associated with local soft tissue irritation, particularly when wire ends protrude or when postoperative micromotion occurs [9,17,20,21]. Non-metallic suture materials lack rigid metallic ends and are less traumatic to surrounding soft tissues, which may reduce postoperative discomfort and implant-related irritation [6,9,17]. Although postoperative pain was not quantitatively assessed in the present case, postoperative inflammatory responses remained mild, as evidenced by a progressive decline in serum C-reactive protein concentrations and only mild, self-limiting local inflammatory changes at the surgical site. Together with the absence of implant-related complications, these findings suggest that FiberWire was well tolerated as a sternal fixation material in this dog.

An additional potential advantage of non-metallic sternal fixation materials is the absence of imaging artifacts during postoperative follow-up [19]. Stainless-steel orthopedic wire may generate metallic artifacts on radiography, computed tomography, or magnetic resonance imaging, which can interfere with assessment of the surgical site or detection of disease recurrence [6,22]. In contrast, FiberWire, as a non-metallic UHMWPE-based suture material, does not produce metal-related imaging artifacts and may therefore facilitate postoperative imaging evaluation in patients undergoing thoracic surgery for neoplastic disease [6]. In the present case, serial thoracic radiographic follow-up demonstrated stable sternal alignment without evidence of dehiscence or implant-related complications, supporting the feasibility of FiberWire for postoperative monitoring following median sternotomy.

This report describes a single clinical case, and the findings should be interpreted with caution. Although no implant-related complications were observed in this case, UHMWPE-based fixation materials may potentially be associated with long-term complications such as infection, foreign body reactions, granuloma formation, or progressive bone-related changes [6]. These potential complications may not be fully captured within the follow-up period of a single case. Although radiographic follow-up was available up to POD 329, the evaluation of a single case limits the ability to fully assess the long-term complication profile associated with this fixation material and technique. Nevertheless, short- to mid-term radiographic follow-up demonstrated stable sternal alignment without evidence of dehiscence or implant-related complications, with findings consistent with progressive sternal healing. Future studies involving patient cohorts and longer follow-up periods are warranted to further evaluate the long-term stability, safety, and clinical applicability of FiberWire as a potential alternative to traditional stainless-steel wire for sternal closure following median sternotomy in dogs.

## 4. Conclusions

This report describes the first documented clinical case of median sternotomy closure using FiberWire following thymectomy. In this case, FiberWire provided stable sternal fixation without postoperative dehiscence or implant-related complications, as confirmed by radiographic follow-up and progressive clinical improvement. These findings support the clinical feasibility of FiberWire as a non-metallic sternal fixation material for use following median sternotomy in dogs. Although this report is limited to a single clinical case, it suggests the potential applicability of FiberWire for sternal closure in canine thoracic surgery. Further studies involving additional case series and longer follow-up periods are warranted to better evaluate its long-term safety and effectiveness.

## Data Availability

The data presented in this study are included in the article. Further inquiries can be directed to the corresponding author.

## Abbreviations

The following abbreviations are used in this manuscript:

CRP	C-reactive protein
CT	Computed tomography
FNA	Fine-needle aspiration
HU	Hounsfield unit
MS	Median sternotomy
POD	Postoperative day
H&E	Hematoxylin and eosin
UHMWPE	Ultra-high-molecular-weight polyethylene
